# In Vitro Antimicrobial Properties and Their Mechanisms in Relation to Reactive Oxygen Species of Canine Platelet-Rich Fibrin

**DOI:** 10.3390/ani13243786

**Published:** 2023-12-08

**Authors:** Ravisa Warin, Preeyanat Vongchan, Witaya Suriyasathaporn, David C. Hall, Ratchadaporn Boripun, Wanna Suriyasathaporn

**Affiliations:** 1 Faculty of Veterinary Medicine, Chiang Mai University, Chiang Mai 50100, Thailand; ravisa_war@cmu.ac.th (R.W.); suriyasathaporn.witaya.y3@f.mail.nagoya-u.ac.jp (W.S.); 2 Department of Medical Technology, Faculty of Associated Medical Sciences, Chiang Mai University, Chiang Mai 50200, Thailand; preeyanat.v@cmu.ac.th; 3 Research Center of Producing and Development of Products and Innovations for Animal Health and Production, Chiang Mai University, Chiang Mai 50100, Thailand; 4 Asian Satellite Campuses Institute, Cambodian Campus, Nagoya University, Nagoya 464-8601, Japan; 5 Faculty of Veterinary Medicine, University of Calgary, Calgary, AB T2N 4Z1, Canada; dchall@ucalgary.ca; 6 Akkhraratchakumari Veterinary College, Walailak University, Nakhon Si Thammarat 80160, Thailand; ratchadaporn.bo@wu.ac.th

**Keywords:** canine platelet-rich fibrin, wound infection, regenerative medicine, dogs, antimicrobial effects, reactive oxygen species

## Abstract

**Simple Summary:**

Bacterial infections can often lead to complications in wound healing. The overuse of antimicrobial drugs can cause antimicrobial resistance, making it necessary to find alternative therapeutic options for wound care. Platelet-rich fibrin (PRF) has been used for tissue regeneration, but until now, its application for antimicrobial activity has been limited to periodontal pathogens. In this study, we aimed to determine the antimicrobial effects of canine PRF (cPRF) against bacteria from the clinical wound of a dog and to investigate the mechanism of its antibacterial activity, which involves the formation of reactive oxygen species (ROS). Our results showed that cPRF had a significant antimicrobial effect against *Escherichia coli* between 4 and 24 h, resulting from the release of ROS. Based on these findings, cPRF, a rich source of growth factors that accelerate the wound healing process, could be used as a biological material for wound infection treatment, benefiting veterinary medicine.

**Abstract:**

Platelet-rich fibrin (PRF), which has been shown to promote wound and bone regeneration, has demonstrated antimicrobial properties against periodontal pathogens. However, in veterinary medicine, no study has determined the antimicrobial effects of canine platelet-rich fibrin (cPRF). Therefore, this study aimed to determine the antimicrobial effect of cPRF against *E. coli* and *S. pseudintermedius* found in dogs’ wounds and against the standard strain *S. aureus*. Additionally, the mechanism of the existing antibacterial activity of cPRF, which involves the formation of reactive oxygen species (ROS), was tested. Blood samples from six dogs were processed for cPRF. The antimicrobial properties of three groups (growth control, cPRF, and drug control) were evaluated at 0.5, 4, 8, and 24 h using a time–kill assay. The killing mechanisms involving ROS were evaluated using horseradish peroxidase (HRP) to suppress ROS production in PRF (PRF-SR). Subsequently, tests for antimicrobial properties and ROS generation were compared to those of the growth control and cPRF groups. The results showed that cPRF had significant antimicrobial properties against *E. coli* but no antimicrobial properties against *S. pseudintermedius*. After the ROS suppression, PRF-SR did not show an antimicrobial property against *E. coli*. Moreover, cPRF-treated bacteria exhibited significantly greater intracellular ROS than PRF-SR. In conclusion, canine PRF showed an antimicrobial effect against *E. coli*, and its antibacterial mechanism was related to releasing ROS.

## 1. Introduction

Skin injuries are common in small animal practice, and wounds classified as Class II to Class IV [1] can lead to complications in wound healing due to bacterial contamination. While the current standard treatment for wounds in veterinary medicine is the use of broad-spectrum antibiotics, the overuse of antibiotics has led to the emergence and rapid spread of antibiotic-resistant bacteria [2,3]. This poses a significant challenge for health practitioners and a massive threat to human and animal health. Research has found that limiting the use of antibiotics for six months on a dairy farm results in a reduction in antibiotic-resistant bacteria [4]. Therefore, it is crucial to introduce alternative treatments for less severe bacterial infections, especially Class II and Class III wounds, in order to minimize the unnecessary use of antibiotics. Doing so will help to mitigate the emergence of antibiotic-resistant bacteria and preserve the effectiveness of antibiotics for future use.

Blood clots are crucial in the natural healing of wounds. They combine platelets and red blood cells, forming a semi-flexible fibrin fiber network [5]. Platelets are believed to be an effective treatment for wound care and play a crucial role in the body’s defense against infection [6]. The natural products of platelets, such as platelet-rich plasma (PRP) and platelet-rich fibrin (PRF), have been utilized in human and veterinary medicine [7,8]. PRF is ideal for tissue regeneration and growth stimulation as it has slower and prolonged releases of growth factors compared to PRP [9], in which PRP requires an anticoagulant, causing delayed wound healing [10]. Additionally, preparing PRP can be complicated and expensive [11]. Therefore, the use of PRF may be more encouraged than PRP. Furthermore, PRP and PRF have demonstrated antimicrobial effects against *Porphyromonas gingivalis* in dentistry [12,13]. In veterinary medicine, PRP has been found to inhibit *Escherichia coli* growth [14], but no study has evaluated the antimicrobial effects of PRF. 

Research has indicated that platelets can generate reactive oxygen species (ROS) that aid in combating microbial infections [15,16]. For example, porcine platelets activated by bacterial lipopolysaccharide (LPS) generate superoxide anions that might be capable of fighting against bacterial infections [17]. However, it was discovered that the ROS generated by equine platelets did not significantly contribute to bacterial eradication [18], and this discrepancy might be due to species differences or different stimuli. *E. coli* is a pathogen that causes various infections in canines, including wound infections [19], and is the most frequently isolated bacterium in surgical wound infections [20]. In contrast, *Staphylococcus pseudintermedius* is the most common bacterium in canine wounds [21,22]. Recent research has reported on the wound-healing property of canine platelet-rich fibrin (cPRF) in vitro and found that cPRF increased fibroblast cell proliferation and induced a significantly faster wound closure than the control [23]. However, no study has yet determined whether cPRF can effectively combat *E. coli* and *S. pseudointermedius* or how it does so. This study explored the in vitro antimicrobial efficacy of canine PRF against bacteria. Time–kill assays were utilized to assess the antimicrobial effect of canine PRF. The study also aimed to determine the mechanism through which canine PRF combats bacteria, subsequently evaluating its existing antibacterial properties by measuring ROS production and examining its antibacterial impact after ROS suppression.

## 2. Materials and Methods

### 2.1. Ethical Approval, Animal Selection, and Study Design 

The animal care and use committee of the Faculty of Veterinary Medicine at Chiang Mai University, Thailand (FVM-CMU-ICUC Ref. No. S27/2563) approved the use of animals for the experiment. The laboratory animal ethics laws and protocols were followed to minimize animal suffering and the number of animals used. The dog owners were informed and consented before their dogs participated in the experiment. 

**Figure 1:**
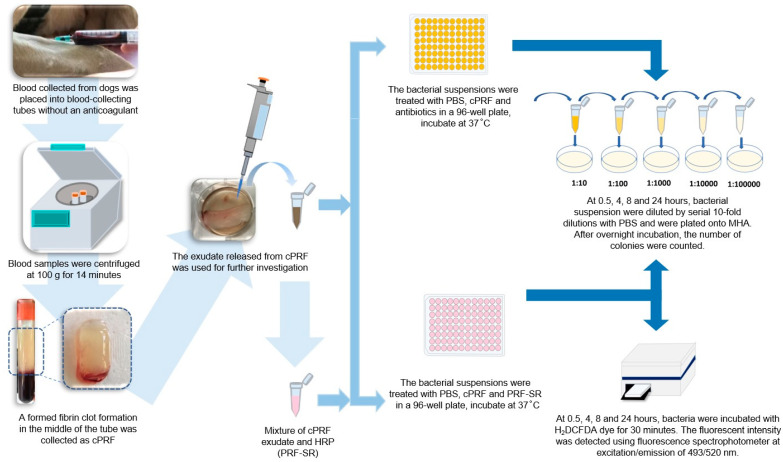
Schematic outline of experiments determining antimicrobial effects and antimicrobial mechanism of canine platelet-rich fibrin (cPRF) using time–kill assay and fluorescent probe 2,7-dichlorodihydrofluorescein diacetate (H_2_DCFDA). HRP: horseradish peroxidase. PBS: phosphate buffer saline.

The study involved six healthy Labrador retriever dogs (three males and three females) from Border Patrol Police Division 33, Chiang Mai, Thailand. As previously reported, the dogs were evaluated for their health status and management [23]. The experiment involved collecting and processing 10 mL of blood from each dog to prepare the canine PRF (cPRF), as described in [23]. The experiment compared the antimicrobial effects of three conditions: (1) growth control using phosphate buffer saline (PBS) (Gibco, Life Technologies Corporation, New York, NY, USA); (2) cPRF; and (3) drug control using antibiotics. The time–kill assay was used at 0.5, 4, 8, and 24 h. To determine the antibacterial mechanism related to the ROS of cPRF, both antimicrobial efficacy and its ROS generation after ROS suppression via horseradish peroxidase (HRP) were tested using a time–kill assay and the fluorescent probe 2,7-dichlorodihydrofluorescein diacetate (H_2_DCFDA), respectively, for the specified bacteria killed by cPRF (Figure 1).

### 2.2. Bacterial Preparation

Bacterial strains used in this study were *S. aureus* (ATCC 29213) (SAs), *E. coli* ATCC 25922 (ECs), clinical isolates of *S. pseudintermedius* (SPc)*,* and *E. coli* (ECc). The two clinical isolates were obtained from cases of canine surgical wound infection submitted to the Chiang Mai University Veterinary Teaching Hospital for routine microbial culture and susceptibility testing, which were identified using automated identification systems (VITEK^®^ 2 COMPACT, bioMerieux, Marcy l’Etoile, France). Subsequently, these isolates confirmed the identification using matrix-assisted laser desorption/ionization time-of-flight mass spectrometry (MALDI Biotyper, Bruker Daltonik GmbH, Bremen, Germany). For antimicrobial susceptibility testing, the minimal inhibitory concentration (MIC) values were used and categorized into S, I, or R according to susceptible, intermediate, or resistant. All isolates were defrosted and cultured in Brain Heart Infusion (BHI) agar (Himedia^®^, Mumbai, India) at 37 °C overnight before use.

### 2.3. Time–Kill Assay

Time–kill assays were conducted with modifications according to Clinical Laboratory Standards Institute (CLSI) guidelines [24,25]. The bacterial suspension was adjusted to a final 5 × 10^5^ CFU/mL concentration. The bacterial suspensions were prepared in a 96-well plate for 200 µL/well volume in a 1:1 ratio. Antibiotics, including Enrofloxacin (Genfloxcin; General Drugs House Co., Ltd., Pathum Thani, Thailand) 0.5 µg/mL, Clindamycin (MACCLINDA; Macrophar Co., Ltd., Bangkok, Thailand) 0.5 µg/mL, Gentamicin (Gentamicin sulfate; T.P. Drug Laboratories Co., Ltd., Bangkok, Thailand) 4 µg/mL, and Clindamycin 0.5 µg/mL, were used as the drug controls for ECc, SPc, ECs, and SAs, respectively, according to CLSI guidelines [24,26]. The incubation was carried out at 37 °C. The assay was performed in duplicate and repeated twice. The bacterial colony numbers were quantified after incubation at 0.5, 4, 8, and 24 h. At the specified time, aliquots of the bacterial suspension were diluted using serial 10-fold dilutions in PBS. Then, a 50 µL sample of each dilution was plated onto Muller–Hinton Agar (MHA) (Himedia^®^, Maharashtra, India). After overnight incubation, the number of viable bacteria was determined by counting the colonies. The number of colonies ranging from 30 to 300 was considered countable.

### 2.4. Antimicrobial Mechanism RELATED to ROS Determination 

The antimicrobial mechanism related to ROS determination was tested for the specified bacteria killed by cPRF according to the time–kill assay result using an enzymatic treatment to suppress superoxide as a ROS. The horseradish peroxidase (HRP) enzyme (Sigma Aldrich, St. Louis, MO, USA), the enzyme for suppressing the peroxide released from the cPRF, at a final concentration of 0.2 mg/mL, was mixed with cPRF exudate at a ratio of 1:1. The solution was sterilized with a sterile syringe filter (Corning^®^, Corning Incorporated, Berlin, Germany) and incubated at 37 °C for 30 min before use as cPRF with suppressed ROS (PRF-SR). The bacterial suspension was treated with PBS as a control, cPRF, and PRF-SR for a total volume of 200 µL/well (ratio 1:1). Numbers of colonies were quantified under a similar condition using time–kill assays as previously described. 

The ROS generations of a control condition using PBS, cPRF, and PRF-SR at 0.5, 4, 8, and 24 h were measured using a fluorescent probe 2,7-dichlorodihydrofluorescein diacetate (H_2_DCFDA) (Invitrogen, Thermo Fisher Scientific, Life Technologies Corporation, Eugene, OR, USA) according to the manufacturer’s instructions and Jyung and colleagues [27], with slight modifications. Briefly, at the desired time points, bacteria were incubated with H_2_DCFDA dye at a final concentration of 10 µM at 37 °C for 30 min. Subsequently, the fluorescent intensity was detected using a fluorescence spectrophotometer (Synergy™ H1, BioTek^®^ Instruments Incorporated, Winooski, VT, USA) at excitation/emission wavelengths of 493/520 nm. The experiment was performed in triplicate. Fluorescent intensity (Relative Fluorescence Units: RFU) was presented as an F/F_0_ value, calculated via the fluorescence of the test (F) divided by the fluorescence of the control at 0.5 h (F_0_).

### 2.5. Statistical Analysis

The data are presented as means and standard errors (SEs). Non-normally distributed data were transformed using a logarithm. As the data were collected from the same dogs, repeated measure analyses were performed using a generalized linear mixed model (Proc mixed, SAS, 2004) to identify group differences. All independent variables, including groups and times, were treated as categorical variables. The number of colonies and F/F_0_ values were dependent variables separately analyzed among groups at 0.5, 4, 8, and 24 h. Least-square means were calculated for all comparisons, and significance was defined at *p* < 0.05. 

## 3. Results

Table 1 presents the results of the antimicrobial susceptibility tests carried out on all the isolates. For SPc isolates that exhibited phenotypic resistance to Oxacillin with a MIC greater than 0.5 µg/mL, Methicillin-resistant *S. pseudintermedius* (MR-SPc) was considered. Both SPc isolates were also resistant to benzylpenicillin, clindamycin, enrofloxacin, marbofloxacin, and trimethoprim/sulfamethoxacin. In contrast, SAs, ECs, and ECc isolates displayed sensitivity to almost all the antibiotics tested.

**Figure 2:**
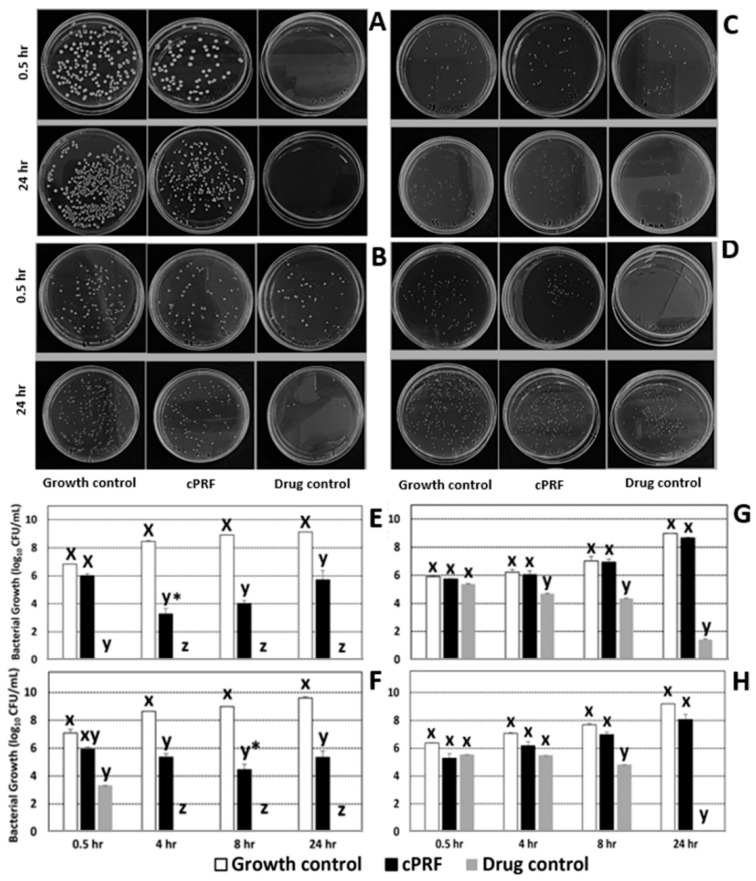
The time–kill assay images were captured for (**A**) *E. coli* ATCC 25922 or ECs; (**B**) *E. coli* clinical isolate or ECc; (**C**) Methicillin-resistant *S. pseudintermedius* clinical isolate or MR-SPc; and (**D**) *S. aureus* ATCC 29213 or SAs for each group, including growth control, cPRF, and drug control. The lower series figure indicates the antimicrobial properties of cPRF, growth control, and drug control against (**E**) ECs; (**F**) ECc; (**G**) MR-SPc; and (**H**) SAs at 0.5, 4, 8, and 24 h. Based on the results of a generalized linear mixed model, the x, y, and z letters indicate differences between groups within the same time at *p* < 0.05, and * indicates differences in the specified time from 0.5 h within cPRF at *p* < 0.05.

Representative time-lapse images of bacterial colonies in the time–kill assays, at 0.5 and 24 h after culturing among different groups, are shown in Figure 2A–D. The results demonstrate that cPRF exhibits a lower bacterial colony count than the growth control, indicating the presence of antimicrobial activity of cPRF in ECs and ECc cultures (Figure 2A,B). The antimicrobial effects of cPRF on all bacteria compared to the control and antibiotics at 0.5, 4, 8, and 24 h are displayed in Figure 2E–H. At 0.5 h, there was no discernible difference in the number of *E. coli* colonies between the growth control and cPRF. However, cPRF had significantly fewer colonies in both *E. coli* strains at 4, 8, and 24 h than the growth control (Figure 2E,F). In comparison, within cPRF, the numbers of bacteria at 4 and 8 h were significantly lower than at 0.5 h for ECs (*p* < 0.0001) and ECc (*p* = 0.03), respectively. There was no antimicrobial effect of cPRF on MR-SPc and SAs (Figure 2G,H). The colonies in the antibiotic group were lower than the control and cPRF at 4, 8, and 24 h for MR-SPc and 8 and 24 h for SAs.

**Figure 3:**
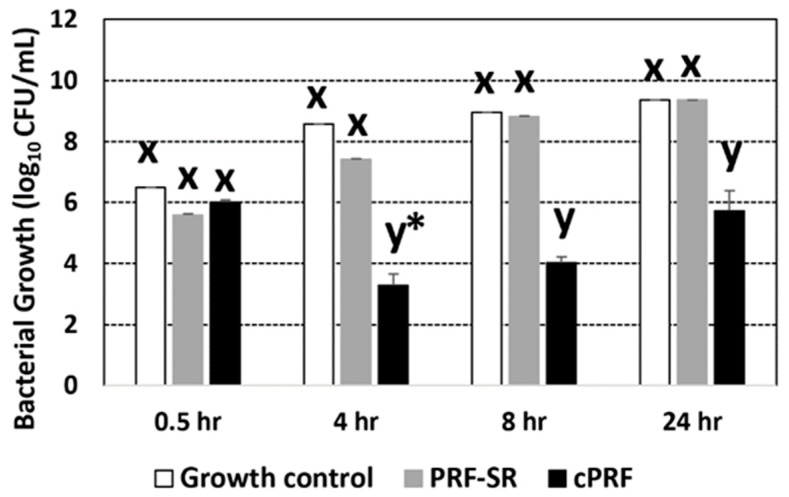
Numbers of colonies of *E. coli* ATCC 25922 culturing under the control condition, PRF-SR as enzymatic treatment of PRF, and cPRF at 0.5, 4, 8, and 24 h (h). Based on the results of a generalized linear mixed model, x and y are different letters indicating the differences between groups within the same time at *p* < 0.05, and * indicates differences of the specified time from 0.5 h within cPRF at *p* < 0.05.

Figure 3 compares the number of colonies of *E. coli* among the growth control, PRF-SR, and cPRF. No difference in bacterial growth was observed among groups at 0.5 h. cPRF showed a significant decrease in the number of colonies at 4, 8, and 24 h of incubation compared with the growth control and PRF-SR. In comparison, within cPRF, the number of bacteria decreased significantly at 4 h compared to 0.5 h. 

**Figure 4:**
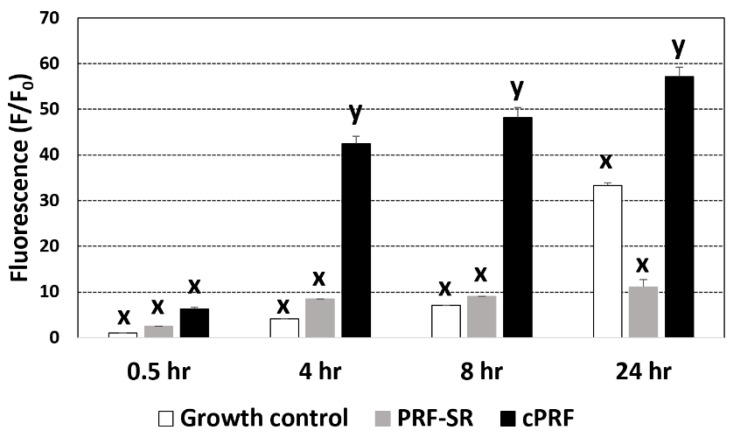
ROS generation in *E. coli* ATCC 25922 culturing under control conditions, cPRF with suppressed ROS (PRF-SR), and cPRF at 0.5, 4, 8, and 24 h (h). Based on the results of a generalized linear mixed model, x and y are different letters indicating differences between groups within the same time at *p* < 0.05.

In Figure 4, the ROS generation of cultures stimulated with ECs was compared among different groups based on their cell fluorescence intensity. At 0.5 h, the fluorescence intensity was similar in all groups. However, at 4, 8, and 24 h, cPRF had a significantly higher fluorescence intensity than the growth control and PRF-SR. There were no significant differences in the fluorescent intensity between the growth control and PRF-SR at any time.

## 4. Discussion

PRF, the new generation of platelet concentrates, has been shown to promote soft tissue wound healing and bone regeneration in both human and veterinary medicine [28,29,30] due to its growth factors that stimulate healing by promoting granulation tissue formation and angiogenesis [31]. Moreover, in dentistry, PRF has been demonstrated to promote bone regeneration in rabbit teeth [32,33]. While previous studies have explored the antimicrobial effects of PRF on periodontal pathogens [12,13,34,35], in veterinary medicine, this study is the first to investigate the antimicrobial effects of PRF produced from canine blood samples against both standard bacteria and bacteria from the clinical wound of a dog. These findings are important, as wound infections are a serious concern. For the identification of SPc isolates, molecular methods are accurate in differentiating *S. pseudintermedius* from the *S. intermedius* group (SIG). However, MALDI-TOF MS has become a highly reliable and rapid tool for discriminating the organisms belonging to this group [36,37]. Similar to *S. aureus* infection, *S. pseudintermedius*, primarily found in animals, is an opportunistic pathogen that causes disease in conditions with impaired skin barriers, such as surgical procedures, immunosuppressive disorders, and atopic dermatitis [37]. Regarding antimicrobial susceptibility, both MR-SPc isolates were classified as multidrug-resistant (MDR) [38], which is increasingly common in small animal veterinary practice [39,40,41,42]. This poses a challenge due to limited antibiotic use and the potential for spreading among animals [43]. 

In this study, cPRF showed a significant decrease in *E. coli* growth at 4, 8, and 24 h compared to the control group. These findings are consistent with a previous study that showed equine platelets from PRP can significantly decrease *E. coli* growth [18]. Interestingly, the present research discovered that cPRF might have longer antimicrobial effects against *E. coli* than PRP, which had no antimicrobial effects after 4 h in a previous study [18]. This suggests that concentrated leukocytes and a three-dimensional fibrin network contained in PRF can facilitate longer antimicrobial mechanisms [44,45]. Long-term antibiotic treatment can induce the mutation of *E.coli* into the MDR *E.coli* strain [46]. As a result, using cPRF as an antimicrobial agent against *E.coli* could reduce antibiotic use and antimicrobial resistance. At 0.5 h, there was no significant difference in *E. coli* growth in the culture with PRF compared to the control group. Burnouf and colleagues found that PRP had antimicrobial properties against *E. coli* after 3 h of incubation [47]. However, our findings showed that cPRF had no antibacterial properties against Methicillin-resistant *S. pseudintermedius* and *S. aureus*. Bayer and colleagues also found that some staphylococcal strains resisted the antimicrobial molecules of platelets [48]. The study of Wu and colleagues revealed that lipoteichoic acid (LTA), a cell wall component of Gram-positive bacteria, can inhibit platelets’ mediator release and platelet–leukocyte aggregation [49]. Thus, platelet response to infection was altered and might relate to the absent antimicrobial effect of cPRF against Gram-positive bacteria. However, a study using PRP from human samples found antimicrobial effects against *S. aureus* [50]. It is possible that the antimicrobial activity of platelet microbiocidal proteins, one of the antimicrobial molecules from platelets, is directly related to staphylococcal strains, in which different strains show different susceptibilities [48], and species differences between humans and animals should be noted. 

Reactive oxygen species (ROS) are involved in wound-healing processes, including cell motility, cytokine signal transduction, and angiogenesis [51]. Human platelets have been shown to release superoxide and hydrogen peroxide, both of which exert antibacterial action [52,53]. However, ROS production by canine PRF has not been documented. Therefore, this study was the first to investigate the involvement of ROS in the antimicrobial properties of canine PRF. After suppressing ROS production, PRF without ROS or PRF-SR could not kill *E. coli* (Figure 3). This result demonstrated that the antibacterial activity of cPRF resulted from ROS. This finding was supported by a previous study that showed that the release of peroxide in PRF involved its antimicrobial properties [54]. The fluorescence intensity of ROS in cPRF was significantly greater than that of ROS-suppressed PRF and the control at 4, 8, and 24 h (Figure 4), supporting our finding that ROS production was related to the antibacterial properties of PRF (Figure 3). This finding also indicates that PRF could stimulate the production of endogenous ROS, causing the killing of bacteria, which has been proposed as a common mechanism whereby bactericidal antibiotics induce bacterial cell death [55]. The pivotal role of ROS and mitochondria in platelet function has recently emerged, regulating platelet activation, aggregation, and recruitment and tuning several cellular signaling pathways (for a review, see [56]). Nicotinamide adenine dinucleotide (phosphate) (NAD(P)H) oxidase (NOX) isoforms are the main sources of ROS in platelets, followed by cyclooxygenase (COX), xanthine oxidase (XO), and mitochondrial respiration [57]. 

## 5. Conclusions

In conclusion, canine platelet-rich fibrin exhibited promising partly antimicrobial results by lowering *E. coli* growth from 4 to 24 h, but not the growth of *S. pseudintermedius*. The present study demonstrated that the bacterial killing mechanism of canine PRF involved the production of reactive oxygen species. Based on these findings, canine PRF, a rich source of the growth factors that accelerate wound healing, can be partly recommended as a biological material for wound infection treatment. Future investigations studying the antimicrobial effects of canine PRF in vivo should be undertaken to confirm these findings. 

## Figures and Tables

**Table 1:** Minimal inhibitory concentration (MIC) and their interpretations as susceptible (S), intermediate (I), and resistant (R) for *E. coli* clinical isolate (ECc), *S. pseudintermedius* clinical isolate (SPc), *E. coli* ATCC 25922 (ECs), and *S. aureus* ATCC 29213 (SAs) determined using automated identification systems (VITEK^®^ 2 COMPACT, bioMerieux, Marcy l’Etoile, France).

	Antibiotics Tested for All Isolates															
Isolates	AK		C		DO		ENR		CN		MAR		F			
ECc 1	≤2	S	≤2	S	8	I	0.5	S	≤1	S	≤0.5	S	≤16	S		
ECc 2	≤2	S	4	S	1	S	0.5	S	≤1	S	1	S	≤16	S		
ECs	≤4	S	≤8	S	≤2	S	-	-	≤1	S	-	-	≤16	S		
SPc 1	≤2	S	≥64	R	8	I	≥4	R	≥16	R	≥4	R	≤16	S		
SPc 2	≤2	S	8	S	≥16	R	≥4	R	8	I	≥4	R	≤16	S		
SAs	≤2	S	≤8	S	0.5	S	-	-	≤1	S	-	-	≤32	S		
	Antibiotics tested for Gram-negative bacteria															
	AMC		AMP		EFT		CL		KF		IPM		N			
ECc 1	4	S	≥32	R	≤1	S	8	S	4	S	≤0.25	S	≤2	S		
ECc 2	4	S	8	S	≤1	S	8	S	16	I	≤0.25	S	≤2	S		
ECs	2/1	S	8	S	-	-	-	-	4	S	≤0.25	S	-	-		
	Antibiotics tested for Gram-positive bacteria															
	P		CVN		DA		FFC		MH		OX		PRA		SXT	
SPc 1	≥0.5	R	≥8	R	≥4	R	≤4	S	8	I	≥4	R	2	R	≥3	R
SPc 2	≥0.5	R	4	I	≥4	R	≤4	S	1	S	≥4	R	1	I	≥3	R
SAs	≥0.5	R	-	-	0.25	S	-	-	0.5	S	≤0.5	S	-	-	≤0.5	S

Amikacin (AK), Amoxicillin/clavulanic acid (AMC), Ampicillin (AMP), Benzylpenicillin (P), Ceftiofur (EFT), Cefalexin (CL), cefovecin (CVN), Cephalotin (KF), Chloramphenicol (C), Clindamycin (DA), Doxycycline (DO), Enrofloxacin (ENR), Florfenicol (FFC), Gentamicin (CN), Imipenem (IPM), Marbofloxacin (MAR), Minocycline (MH), Nitrofurantoin (F), Neomycin (N), Oxacillin (OX), Pradofloxacin (PRA), Trimetroprim/sulfamethoxacin (SXT).

## Data Availability

The data presented in this study are available in article.
